# Association between average triglyceride glucose-body mass index and risk of hypertension in middle-aged and elderly Chinese: A study based on Chinese CHARLS cohort data

**DOI:** 10.1371/journal.pone.0337710

**Published:** 2025-12-04

**Authors:** Chuanlei Zheng, Yanhong Liu, Shaobo Zeng, Sisi Xie, Xuanning Luo, Qingfeng Wu

**Affiliations:** 1 School of Public Health and Health Management, Gannan Medical University, Ganzhou, China; 2 Key Laboratory of Prevention and Treatment of Cardiovascular and Cerebrovascular Diseases, Ministry of Education, Gannan Medical University, Ganzhou, China; 3 School of Public Health, Shandong Second Medical University, Weifang, China; Instituto Nacional de Cardiologia Ignacio Chavez, MEXICO

## Abstract

**Objective:**

This study aimed to investigate the relationship between cumulative average triglyceride glucose-body mass index (TyG-BMI) and the risk of developing hypertension among middle-aged and elderly population.

**Methods:**

Data were sourced from the 2012 and 2015 China Health and Retirement Longitudinal Study. The population was stratified into four exposure levels based on the quartiles of average TyG-BMI. Logistic regression analyses were employed to assess the associations between varying levels of average TyG-BMI and the risk of hypertension. Additionally, restricted cubic spline regression models were utilized to delineate the dose-response relationship between average TyG-BMI and hypertension. The contributions of fasting plasma glucose, triglyceride, and body mass index were quantified using weighted quantile sum regression to comprehensively elucidate the role of each component of TyG-BMI.

**Results:**

A total of 2,841 participants aged 45 years and older were included in this study, of whom 1,302 (45.83%) were male, with a mean age of 57.63 ± 8.39 years at baseline. Throughout the follow-up period, 704 (24.78%) participants developed new onset hypertension. After adjusting for confounding variables, higher average TyG-BMI levels (from two measurements) were associated with an increased risk of developing hypertension (*Q*2: *OR* = 1.37, 95% *CI* = 1.04–1.80; *Q*3: *OR* = 1.93, 95% *CI* = 1.46–2.56; *Q*4: *OR* = 2.71, 95% *CI* = 2.01–3.66). The results from the restricted cubic spline regression indicated a linear association between average TyG-BMI and the risk of developing hypertension (*P* for association < 0.001, *P* for nonlinear = 0.078). Weighted quantile sum regression revealed that BMI was a significant component of TyG-BMI, with weights of 0.666 and 0.769 in 2012 and 2015, respectively.

**Conclusion:**

Average TyG-BMI emerged as an independent risk factor for new onset hypertension, exhibiting a linear correlation between the two variables. Therefore, long-term monitoring of changes in TyG-BMI should have been a critical component of hypertension prevention strategies.

## Introduction

Hypertension is a significant risk factor for cardiovascular disease and premature death, imposing a substantial economic burden on various countries [1,2], particularly in low-income and middle-income nations [3]. Mei Zhang et al. [4] conducted an analysis of three national surveys spanning from 2004 to 2018. Their findings indicated that the standardized prevalence of hypertension among Chinese adults increased from 20.8% in 2004 to 29.6% in 2010, followed by a decrease to 24.7% in 2018. Despite this downward trend, the prevalence remains elevated compared to the figures recorded in 2004. The prevalence of hypertension will continue to rise with the increasing aging of Chinese society, leading to more prominent adverse effects caused by hypertension.

Currently, hypertension is not curable. The early identification of high-risk groups and timely interventions to control their risk factors are crucial prevention and management strategies for hypertension. Research has demonstrated that insulin resistance is a significant predictive factor for the development of hypertension [5]. However, the process of detecting this indicator is complex, time-consuming, and costly, which limits its clinical application [6]. In recent years, studies have indicated that the triglyceride-glucose index (TyG) can serve as a novel surrogate marker for evaluating insulin resistance [7,8]. Given the high availability and low cost of the biomarkers involved, this approach is more conducive to clinical promotion and application [9]. Obesity remains the most critical acquired factor contributing to insulin resistance, yet the TyG has not accounted for the impact of obesity on this condition [10]. When combined with obesity indicators such as body mass index (BMI), waist circumference, and waist-to-height ratio, the efficacy of evaluating insulin resistance could be enhanced [11,12]. One study compared the associations of TyG, triglyceride glucose-body mass index (TyG-BMI), triglyceride glucose-waist circumference, and triglyceride glucose-waist-height ratio with insulin resistance. The results indicated that TyG-BMI outperformed the other parameters in predicting insulin resistance, further supporting its potential as a surrogate marker for assessing insulin resistance in clinical settings [13]. Additionally, other studies have shown that TyG-BMI demonstrates strong predictive ability for cardiovascular disease, heart failure, and metabolic syndrome [14–16].

Additionally, studies have indicated that the TyG-BMI is a more effective predictor of hypertension compared to the TyG [17,18]. Danying Deng et al [19] revealed that for each one standard deviation increase in TyG-BMI, the risk of developing hypertension escalated by a factor of 1.51. Nonetheless, the majority of existing studies rely on cross-sectional research designs, with limited longitudinal evidence examining the cumulative impact of TyG-BMI on hypertension incidence. Consequently, this study utilized data from the China Health and Retirement Longitudinal Study (CHARLS) conducted in 2012 and 2015 to assess the relationship between average TyG-BMI and hypertension risk among middle-aged and elderly individuals in China.

## Methods

### Study population

**Fig 1:**
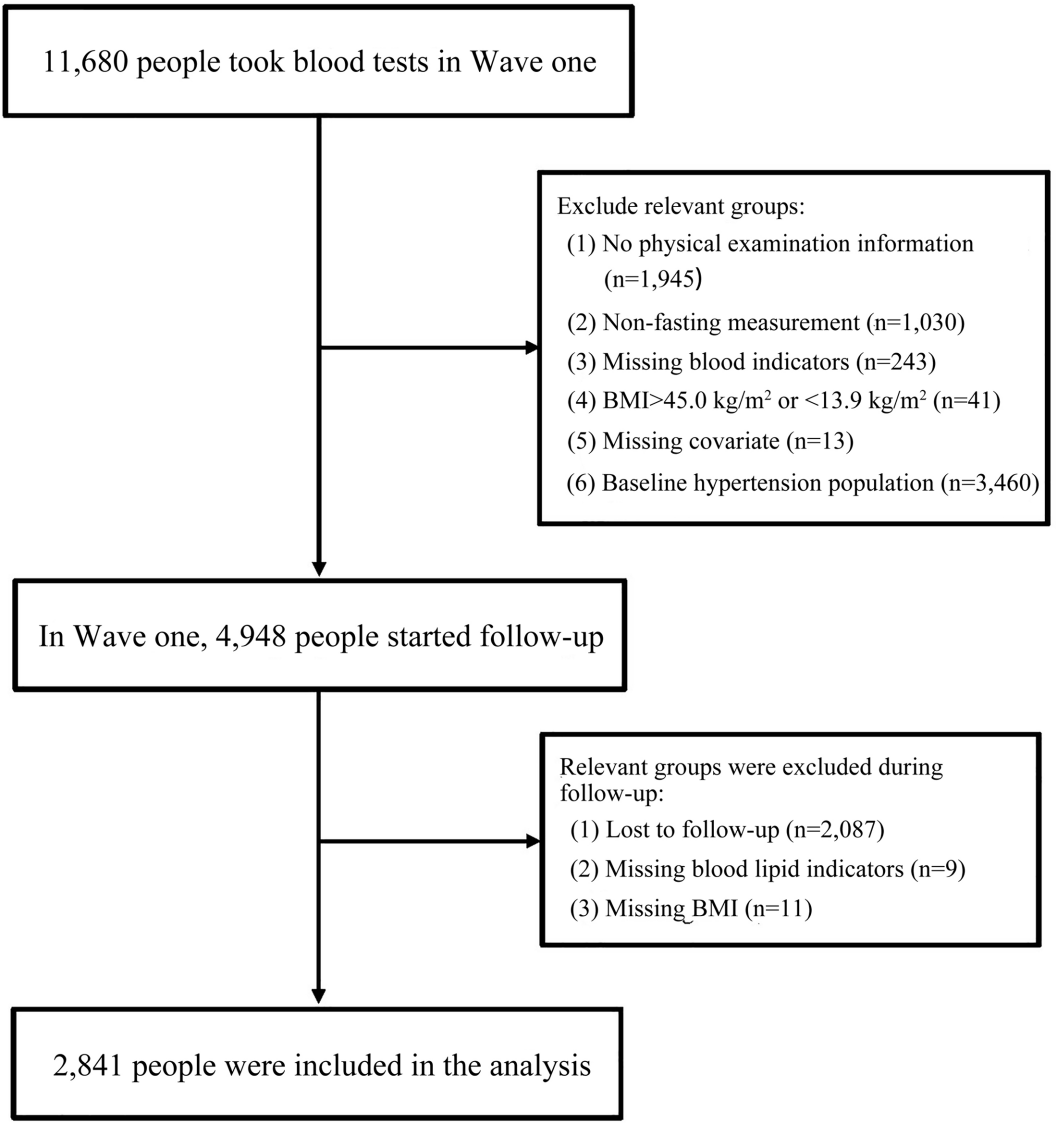
Inclusion and exclusion criteria for study population. CHARLS, China Health and Retirement Longitudinal Study; BMI, body mass index.

The data for this study were derived from the CHARLS conducted in 2011 and 2015 [20,21]. The CHARLS collected high-quality data on families and individuals aged 45 and older in China to analyze issues related to population aging and to promote interdisciplinary research in this field. The national baseline survey (Wave 1) was carried out from 2011 to 2012, encompassing 150 county-level units, 450 village-level units, and approximately 17,000 individuals across 10,000 households. These samples were tracked every two to three years, with subsequent waves occurring in 2013 (Wave 2), 2015 (Wave 3), 2018 (Wave 4), and 2020 (Wave 5). Only Wave 1 (n = 11,847) and Wave 3 (n = 13,420) collected blood indicators from participants [22]; thus, a total of 11,847 participants with collected blood indicators were included in this study at baseline. The inclusion criteria were: (1) aged = 45 or older; (2) willingness to participate in the study and informed consent provided; (3) no mental illness and ability to cooperate with the investigation. The exclusion criteria were: (1) missing data on relevant variables; (2) non-fasting blood collection indicators; (3) BMI > 45.0 kg/m² or < 13.9 kg/m²; (4) pre-existing hypertension at baseline; (5) loss to follow-up; (6) patients with severe heart, brain, kidney, or other serious diseases and pregnant women. Based on these criteria, a total of 2,841 individuals were ultimately included in the analysis.(Fig 1). The Biomedical Ethics Review Board of Peking University approved the collection of CHARLS data (IRB00001052–11015). Before the survey commenced, professionally trained interviewers provided all participants with comprehensive explanations of the study’s objectives, procedures, potential risks, anticipated benefits, and measures for data confidentiality. Each participant signed a written informed consent form that was approved by the ethics committee. For individuals unable to participate in person due to physical limitations, family members were allowed to respond on their behalf.

### Average TyG-BMI assessment

In this study, the exposure was defined as the average TyG-BMI, which was calculated as the arithmetic mean of the TyG-BMI values from 2012 and 2015. It is crucial to emphasize that this calculation represents a simple average across the two time points, rather than a time-weighted integral of cumulative exposure. TyG-BMI was calculated from triglyceride (TG), fasting plasma glucose (FPG) and BMI. The TyG-BMI was calculated by the formula $\begin{array}{c} ln[TG(mg/dL)\times FPG(mg/dL)/2]\times BMI(kg/m^{2}) \end{array}$ [23]. We calculated the average TyG-BMI with reference to the cumulative average TyG change formula [23,24]: $\begin{array}{c} ({TyG-BMI}_{2012}+{TyG-BMI}_{2015})/time\left(2015-2012\right) \end{array}$, the BMI was calculated by the formula $\begin{array}{c} \frac{weight(kg)}{{height}^{2}(m^{2})} \end{array}$.

### Covariate measure

Trained interviewers utilized structured questionnaires to gather demographic and health-related information. The demographic data encompassed age, sex, residence, marital status, and education. Health-related information included self-rated health status, smoking status, drinking status, and self-reported medical history, specifically regarding dyslipidemia and diabetes. Laboratory tests conducted comprised total cholesterol (TC), low-density lipoprotein cholesterol (LDL-C), high-density lipoprotein cholesterol (HDL-C), and glycosylated hemoglobin (HbA1c).

### Relevant indicators and diagnostic criteria

The primary outcome of this study was the development of hypertension. The diagnostic criteria for hypertension include a systolic blood pressure of ≥140 mmHg and/or a diastolic blood pressure of ≥90 mmHg, measured without the use of antihypertensive medications, or through self-reported hypertension [25]. Dyslipidemia was diagnosed when TC ≥ 240 mg/dL, TG ≥ 150 mg/dL, LDL-C ≥ 160 mg/dL, and HDL-C ≤ 40 mg/dL, without the use of lipid-lowering medications. Additionally, the presence of one or more of these four indicators was required for a diagnosis of abnormal or self-reported dyslipidemia [26]. Type 2 diabetes was defined as a fasting blood glucose level of ≥126 mg/dL or self-reported diabetes, again without the use of antidiabetic medications [27].

### Statistical analyses

The study subjects were divided into four groups (*Q*1-*Q*4) based on the quartiles of average TyG-BMI. Categorical variables were represented by constituent ratios or rates, and *χ*^*2*^ test was employed to compare differences between groups. For continuous variables with a normal distribution, $\overset{―}{x}\pm s$ was used for description, and differences between groups were analyzed using ANOVA test. For continuous variables with a non-normal distribution, the median (*P*_50_) along with the interquartile range (*P*_25_, *P*_75_) was reported, and differences between groups were assessed using the Wilcoxon rank sum test. Linear regression was employed to assess multicollinearity among variables. Variance inflation factor (VIF) > 5 or tolerance < 0.1 indicated the presence of multicollinearity. After fully adjusting for covariates, the logistic regression model was used to analyze the association between average TyG-BMI and the risk of hypertension. The bayesian information criterion (BIC) and Akaike information criterion (AIC) of different nodes were comprehensively compared to determine the nodes of average TyG-BMI, and the restricted cubic spline(RCS) model was constructed to explore the dose-response relationship between accumulated TyG-BMI and the risk of hypertension.

To further investigate the association between average TyG-BMI and the risk of hypertension, a series of subgroup analyses were conducted based on potential risk factors, including sex, age groups, place of residence, education background, marital status, and other relevant factors. After splitting the data into a 40% training set and a 60% validation set, weighted quantile sum (WQS) regression model and Bootstrap resampling processes (1,000 iterations) were conducted to determine the specific contributions of TyG-BMI components to the overall impact [23,28]. A two-sided test was employed with a significance level of *α* = 0.05. All statistical analyses were conducted using SPSS version 14.0 and R version 4.3.2.

## Results

### Baseline characteristics of participants

A total of 2,841 eligible subjects were included in this study, comprising 1,302 males (45.83%) and 1,539 females (54.17%). The average age at baseline was 57.63 ± 8.39 years. The TyG-BMI_2012_ and TyG-BMI_2015_ were 197.69 ± 36.01 and 216.38 ± 39.66, respectively, with a average TyG-BMI of 621.11 ± 108.42. Statistical differences were observed among the four groups (*Q*1-*Q*4) regarding sex, age, residence, education, marital status, health status, smoking status, drinking status, history of dyslipidemia, history of diabetes, FPG, HbA1c, TC, LDL-C, HDL-C, TG, BMI, and TyG (*P* < 0.05). (Table 1).

**Table 1:** Baseline characteristics by average TyG-BMI quartiles.

Characteristic	Overall (n = 2841)	Quartiles of the average TyG-BMI				*χ*^2^/*F*/*Z*	*P*
		*Q*1[370.74,541.29]	*Q*2(541.29,611.86]	*Q*3(611.86,686.09]	*Q*4(686.9,1124.09]		
Sex						114.28^a^	<0.001^***^
Male	1,302 (45.83)	422 (59.35)	364 (51.27)	271 (38.17)	245 (34.51)		
Female	1,539 (54.17)	289 (40.65)	346 (48.73)	439 (61.83)	465 (65.19)		
Residence						30.99^a^	<0.001^***^
Rural	2,446 (86.10)	641 (90.15)	632 (89.01)	595 (83.80)	578 (81.41)		
Other	395 (13.90)	70 (9.85)	78 (10.99)	115 (16.20)	132 (18.59)		
Education						20.45^a^	0.002^**^
Illiteracy	780 (27.46)	201 (28.27)	201 (28.31)	205 (28.87)	173 (24.37)		
Primary school	1,167 (41.08)	323 (45.43)	294 (41.41)	266 (37.46)	284 (40.00)		
Middle school and above	894 (31.47)	187 (26.30)	215 (30.28)	239 (33.66)	253 (35.63)		
Marital status						14.46^a^	0.002^**^
Married	2,593 (91.27)	630 (88.61)	644 (90.70)	650 (91.55)	669 (94.23)		
Other	248 (8.73)	81 (11.39)	66 (9.30)	60 (8.45)	41 (5.78)		
Health status						19.69^a^	0.003^**^
Good	678 (23.86)	139 (19.55)	174 (24.51)	180 (25.35)	185 (27.21)		
General	1,411 (40.16)	354 (49.79)	336 (47.32)	356 (50.14)	365 (53.68)		
Not good	752 (26.47)	218 (30.66)	200 (28.17)	174 (24.51)	160 (11.11)		
Smoking status						120.96^a^	<0.001^***^
Non-smoke	1,765 (62.13)	336 (47.23)	419 (59.01)	491 (69.15)	519 (73.10)		
Smoking	1,076 (37.87)	375 (52.74)	291 (40.99)	219 (30.85)	191 (26.90)		
Drinking status						31.29^a^	<0.001^***^
Non-drink	1,867 (65.72)	416 (58.51)	452 (63.66)	501 (70.56)	498 (70.14)		
Drinking	974(34.28)	295 (41.49)	258 (36.34)	209 (29.44)	212 (29.86)		
History of dyslipidemia						364.81^a^	<0.001^***^
No	1,641 (57.8)	561 (78.9)	485 (68.3)	367 (51.7)	228 (32.1)		
Yes	1,200 (42.2)	150 (21.1)	225 (31.7)	343 (48.3)	482 (67.9)		
History of diabetes						75.68^a^	<0.001^***^
No	323 (11.4)	662 (93.1)	657 (92.5)	630 (88.7)	569 (80.1)		
Yes	2,518 (88.6)	49 (6.9)	53 (7.5)	80 (11.3)	141 (19.9)		
Age (year)	57.63 ± 8.39	60.36 ± 8.89	58.25 ± 8.43	56.65 ± 7.76	55.27 ± 7.56	49.62^b^	<0.001^***^
FPG (mg/dL)	106.60 ± 30.35	100.40 ± 19.97	103.08 ± 21.48	105.87 ± 27.93	117.06 ± 43.46	30.44^b^	<0.001^***^
HbA1c (%)	5.23 ± 0.75	5.13 ± 0.55	5.14 ± 0.56	5.22 ± 0.76	5.44 ± 1.00	19.84^b^	<0.001^***^
TC (mg/dL)	191.37 ± 36.86	184.87 ± 36.06	186.75 ± 35.45	193.90 ± 36.93	199.98 ± 37.07	25.55^b^	<0.001^***^
LDL-C (mg/dL)	115.77 ± 33.35	109.90 ± 31.40	114.42 ± 31.60	120.25 ± 32.69	118.53 ± 36.57	14.41^b^	<0.001^***^
HDL-C (mg/dL)	52.24 ± 15.30	60.13 ± 16.39	54.35 ± 14.72	50.79 ± 13.04	43.68 ± 11.80	176.39^b^	<0.001^***^
BMI (kg/m^2^)	22.97 ± 3.39	19.30 ± 1.53	21.79 ± 1.33	23.76 ± 1.47	27.04 ± 2.74	1,872.01^b^	<0.001^***^
TyG	8.58 ± 0.61	8.21 ± 0.44	8.43 ± 0.48	8.62 ± 0.50	9.06 ± 0.67	288.21^b^	<0.001^***^
TyG-BMI_2012_	197.69 ± 36.01	158.37 ± 13.28	183.31 ± 11.38	204.54 ± 12.62	244.59 ± 26.86	2,668.76^b^	<0.001^***^
TyG-BMI_2015_	216.38 ± 39.66	171.22 ± 14.47	201.10 ± 11.87	225.88 ± 13.87	267.41 ± 27.91	3,034.55^b^	<0.001^***^
TG (mg/dL)	97.35 [70.36,139.83]	72.57 [57.53,92.93]	89.39 [66.15,118.81]	104.87 [77.00,144.26]	146.02 [104.21,213.29]	499.95^c^	<0.001^***^

a, *χ*^2^ test, *n*(%); b, ANOVA test, $\overset{―}{x}\pm s$; c, Wilcoxon rank sum test, *P*_50_ (*P*_25_, *P*_75_).

FPG, Fasting plasma glucose; HbA1c, Glycosylated hemoglobin; TC, Total cholesterol; LDL-C, Low-density lipoprotein cholesterol; HDL-C, High-density lipoprotein cholesterol; BMI, Body mass index; TyG, Triglyceride-glucose index; TyG-BMI, Triglyceride glucose-body mass index; TG, Triglyceride; Q1, Quartile 1; Q2, Quartile 2; Q3, Quartile 3; Q4, Quartile 4.

*P < 0.05, **P < 0.01, ***P < 0.001.

### Incidence of hypertension

**Fig 2:**
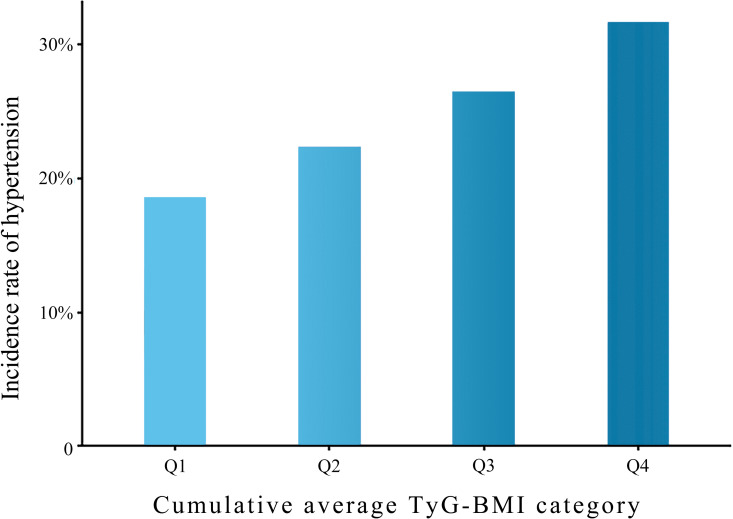
Incidence rates of hypertension categorized by quartiles of the average TyG-BMI. Q1, Quartile 1; Q2, Quartile 2; Q3, Quartile 3; Q4, Quartile 4; TyG-BMI, triglyceride glucose-body mass index.

A total of 704 study subjects (24.78%) developed new onset hypertension during the follow-up period. The prevalence of hypertension exhibited a gradual increase across the different quartiles of average TyG-BMI. The incidence of hypertension in the four groups was 131 (18.42%), 150 (21.13%), 185 (26.06%), and 238 (33.52%) (Fig 2).

### Logistic regression analysis of different average TyG-BMI quartiles and risk of hypertension

Collinearity diagnostic results indicated that the VIF for TC and LDL-C exceeded 5, leading to the exclusion of this factor when adjusting for covariates. The results of univariate logistic regression analysis revealed that, compared to *Q*1, both *Q*3 and *Q*4 exhibited a significantly higher risk of developing hypertension (*Q*3: *OR*=1.56, 95%*CI* = 1.21–2.01; *Q*4: *OR*=2.23, 95%*CI* = 1.75–2.85). No significant association was found between *Q*2 and the risk of hypertension (*P *> 0.05). After adjusting for covariates such as sex, age, residence, and education, logistic regression analysis demonstrated that higher levels of average TyG-BMI (*Q*2-*Q*4) were associated with an increased risk of hypertension (*Q*2: *OR*=1.37, 95%*CI* = 1.04–1.80; *Q*3: *OR*=1.93, 95%*CI* = 1.46–2.56; *Q*4: *OR*=2.71, 95%*CI* = 2.01–3.66) (Table 2 and S1 Table in S1 Appendix).

**Table 2:** Association between different average TyG-BMI quartiles and the incidence of hypertension.

	Unadjusted	*P*	Model 1^a^	*P*	Model 2^b^	*P*
	*OR* (95%*CI*)		*OR* (95%*CI*)		*OR* (95%*CI*)	
Average TyG-BMI						
*Q*1	Reference [370.74-541.29]		Reference [370.74-541.29]		Reference [370.74-541.29]	
*Q*2	1.19 (0.91-1.54)	0.201	1.32 (1.01-1.72)	0.044	1.37 (1.04-1.80)	0.026^*^
*Q*3	1.56 (1.21-2.01)	<0.001^***^	1.93 (1.48-2.51)	<0.001^***^	1.93 (1.46-2.56)	<0.001^***^
*Q*4	2.23 (1.75-2.85)	<0.001^***^	2.93 (2.25-3.82)	<0.001^***^	2.71 (2.01-3.66)	<0.001^***^

a: Adjust sex, age, residence, education, marital status, health status, smoking status, and drinking status.

b: Adjust the variables in Model 1: history of dyslipidemia, history of diabetes, HDL-C, and HbA1c.

TyG-BMI, Triglyceride glucose-body mass index; OR, Odds ratio; CI, Confidence interval; Q1, Quartile 1; Q2, Quartile 2; Q3, Quartile 3; Q4, Quartile 4; HbA1c, Glycosylated hemoglobin; HDL-C, High-density lipoprotein cholesterol.

*P < 0.05, **P < 0.01, ***P < 0.001.

### Dose-response relationship between average TyG-BMI and risk of hypertension

**Fig 3:**
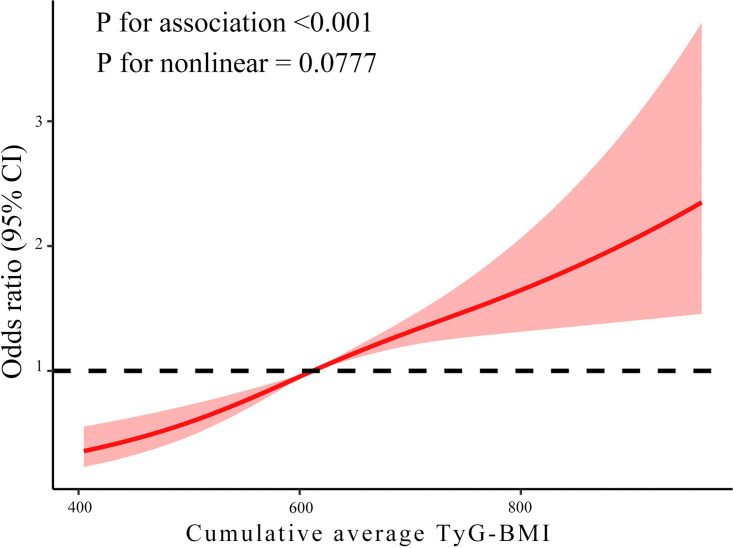
Dose-response relationship between average TyG-BMI and risk of hypertension. The model was adjusted for sex, age, residence, education, marital status, health status, smoking status, drinking status, history of dyslipidemia, history of diabetes, HDL-C, and HbA1c. TyG-BMI, Triglyceride glucose-body mass index; CI, Confidence interval; OR, Odds ratio; HbA1c, Glycosylated hemoglobin; HDL-C, High-density lipoprotein cholesterol.

After comprehensively comparing the BIC and AIC across various nodes, the RCS model was ultimately constructed with three nodes at *P*_10_, *P*_50_, and *P*_90_ of the average TyG-BMI to investigate the relationship between average TyG-BMI and the incidence of hypertension. The dose-response analysis indicated a linear correlation between average TyG-BMI and the risk of hypertension (*P* for association < 0.001, *P* for nonlinearity = 0.078). Notably, when average TyG-BMI exceeded 613 (*OR* = 1.003, 95%*CI *= 1.002–1.003), the risk of hypertension significantly increased (Fig 3 and S2 Table in S1 Appendix).

### Subgroup analyses

To further investigate the association between average TyG-BMI and the risk of hypertension, a series of subgroup analyses were conducted based on potential risk factors. After adjusting for confounding variables, none of the subgroup factors altered the association between average TyG-BMI and the incidence of hypertension, and no interaction between average TyG-BMI and the subgroup variables was observed (*P* for interaction > 0.05) (Table 3).

**Table 3:** Association between average TyG-BMI and different factor stratification on the risk of hypertension.

Subgroup	Quartiles of the average TyG-BMI^a^, *OR* (95% *CI*)				*P* for interaction
	*Q*1	*Q*2	*Q*3	*Q*4	
Sex					0.208
Male	Reference [372.35-541.29]	1.10 (0.76-1.58)	1.93 (1.30-2.85)	2.61 (1.67-4.06)	
Female	Reference [370.74-541.06]	1.87 (1.20-2.91)	2.14 (1.39-3.30)	3.10 (1.99-4.83)	
Age					0.452
<60	Reference [375.71-541.29]	1.47 (0.85-2.55)	2.02 (1.18-3.44)	2.98 (1.73-5.12)	
≥60	Reference [370.74-540.47]	1.33 (0.96-1.83)	1.83 (1.31-2.56)	2.46 (1.69-3.58)	
Residence					0.153
Rural	Reference [370.74-541.29]	1.35 (1.01-1.81)	1.77 (1.31-2.39)	2.80 (2.03-3.87)	
Other	Reference [396.39-540.26]	1.53 (0.61-3.88)	3.50 (1.48-8.30)	2.76 (1.12-6.78)	
Education					0.677
Illiteracy	Reference [380.31-541.06]	1.85 (1.11-3.07)	2.20 (1.31-3.71)	4.46 (2.51-7.92)	
Primary school	Reference [370.74-541.20]	1.17 (0.77-1.79)	1.98 (1.29-3.05)	2.14 (1.35-3.40)	
Middle school and above	Reference [372.35-541.29]	1.56 (0.73-2.16)	1.66 (0.96-2.89)	2.34 (1.32-4.15)	
Marital status					0.763
Married	Reference [370.74-541.29]	1.35 (1.00-1.81)	1.98 (1.47-2.67)	2.84 (2.07-3.92)	
Other	Reference [392.08-541.06]	1.62 (0.73-3.57)	1.46 (0.62-3.43)	1.58 (0.61-4.15)	
Health status					0.252
Good	Reference [414.25-541.20]	0.81 (0.43-1.52)	1.63 (0.88-3.00)	2.15 (1.06-4.37)	
General	Reference [372.35-540.84]	1.62 (1.09-2.41)	2.13 (1.42-3.22)	3.59 (2.34-5.53)	
Not good	Reference [370.74-541.29]	1.55 (0.94-2.54)	1.93 (1.14-3.25)	2.00 (1.16-3.44)	
Smoking status					0.428
Non-smoke	Reference [370.74-541.29]	1.22 (0.83-1.80)	1.96 (1.29-2.98)	2.44 (1.50-3.95)	
Smoking	Reference [372.35-541.14]	1.63 (1.09-2.45)	2.13 (1.43-3.18)	3.11 (2.06-4.68)	
Drinking status					0.409
Non-drink	Reference [373.20-541.06]	1.15 (0.71-1.85)	2.24 (1.34-3.75)	2.25 (1.25-4.04)	
Drinking	Reference [370.74-541.29]	1.45 (1.03-2.04)	1.83 (1.30-2.58)	2.80 (1.96-4.01)	
History of dyslipidemia					0.461
No	Reference [370.74-541.29]	1.29 (0.92-1.80)	2.10 (1.47-3.00)	3.52 (2.30-5.38)	
Yes	Reference [429.18-541.20]	1.54 (0.92-2.58)	1.71 (1.04-2.81)	2.27 (1.39-3.70)	
History of diabetes					0.410
No	Reference [370.74-541.29]	1.40 (1.05-1.87)	2.02 (1.51-2.71)	2.68 (1.95-3.69)	
Yes	Reference [413.97-540.84]	1.01 (0.34-2.99)	1.26 (0.44-3.61)	2.84 (1.02-7.90)	

a: Adjusted for sex, age, residence, education, marital status, health status, smoking status, drinking status, history of dyslipidemia, history of diabetes, HDL-C, HbA1c.

TyG-BMI: triglyceride glucose-body mass index; OR: odds ratio; CI: confidence interval; Q1, Quartile 1; Q2, Quartile 2; Q3, Quartile 3; Q4, Quartile 4; HbA1c, Glycosylated hemoglobin; HDL-C, High-density lipoprotein cholesterol.

### WQS analyses

**Fig 4:**
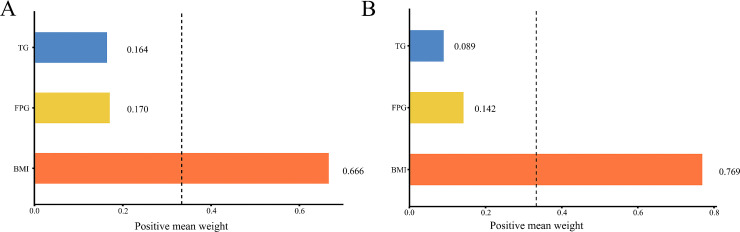
Estimated weights assigned to TyG-BMI with the WQS model. The model was adjusted for sex, age, residence, education, marital status, health status, smoking status, drinking status, history of dyslipidemia, history of diabetes, HDL-C, and HbA1c. TyG-BMI, Triglyceride glucose-body mass index; WQS, Weighted quantile sum FPG, Fasting plasma glucose; BMI, Body mass index; TG, Triglyceride; HbA1c, Glycosylated hemoglobin; HDL-C, High-density lipoprotein cholesterol.

WQS regression model was conducted to assess the overall effect of TyG-BMI on the incidence of hypertension, as well as the contribution of each component (TG, FPG, and BMI) to this overall effect. After adjusting for potential confounding factors, the results indicated that BMI emerged as the primary contributor at both baseline and the conclusion of the follow-up, with weights of 0.666 and 0.769, respectively. The dominant role of BMI in the WQS model suggests that this composite index may offer limited incremental value. Consequently, this study conducted a comparative analysis of the predictive performance of BMI and TyG-BMI using Receiver Operating Characteristic (ROC) curves. The results indicated that TyG-BMI (AUC = 0.594) demonstrated superior predictive efficacy compared to BMI alone (AUC = 0.582). (Fig 4 and S2 Fig in S1 Appendix).

### Sensitivity analysis

This study examined the association between average TyG-BMI, treated as a continuous variable, and the risk of hypertension. Due to the minimal change in effect size per unit of average TyG-BMI, we standardized this variable to assess its effects based on changes in standard deviation. After adjusting for covariates, the results indicated that for each standard deviation increase in average TyG-BMI, the risk of hypertension increased by 43% (*OR*=1.43, 95% *CI* = 1.29–1.58). Furthermore, To avoid reverse causation (i.e., early onset of hypertension may lead to changes in lifestyle or treatment, thereby affecting TyG-BMI), we excluded cases of hypertension from 2013 through sensitivity analysis. A comparison with Q1 revealed that both Q3 and Q4 were associated with an elevated risk of hypertension (*Q*3: *OR*=1.79, 95% *CI* = 1.22–2.63; *Q*4: *OR*=2.41, 95% *CI* = 1.62–3.61). Notably, no association was found between *Q*2 and the risk of hypertension (*P* > 0.05). Next., adjusting for HDL-C and HbA1c, which are closely related to insulin resistance, may constitute over-adjustment and potentially attenuate the observed effects. Therefore, this study further provides the results of the minimally adjusted model for robustness comparison. The study results indicate that, compared to *Q*1, both *Q*2 and *Q*4 are associated with an increased risk of hypertension (*Q*2: *OR*=1.37, 95% *CI* = 1.04–1.80; *Q*3: *OR*=1.93, 95% *CI* = 1.46–2.56; *Q*4: *OR*=2.71, 95% *CI* = 2.01–3.67). Finally, Due to severe multicollinearity (VIF > 5) between TC, LDL-C, and other variables, these two variables were excluded from the primary analysis. However, this approach may overlook critical lipid information. Consequently, we retained these variables in the sensitivity analysis. The results indicated that, compared to the Q1 levels, the average TyG-BMI in the second to fourth quartiles (*Q*2-*Q*4) was associated with an increased risk of hypertension (*Q*2: *OR*=1.36, 95% *CI* = 1.03–1.79; *Q3*: *OR*=1.93, 95% *CI* = 1.45–2.56; *Q4*: *OR*=2.79, 95% *CI* = 2.05–3.80).These findings are consistent with the primary analysis (S3 – S6 Tables and S1 Fig in S1 Appendix).

## Discussion

**Fig 5:**
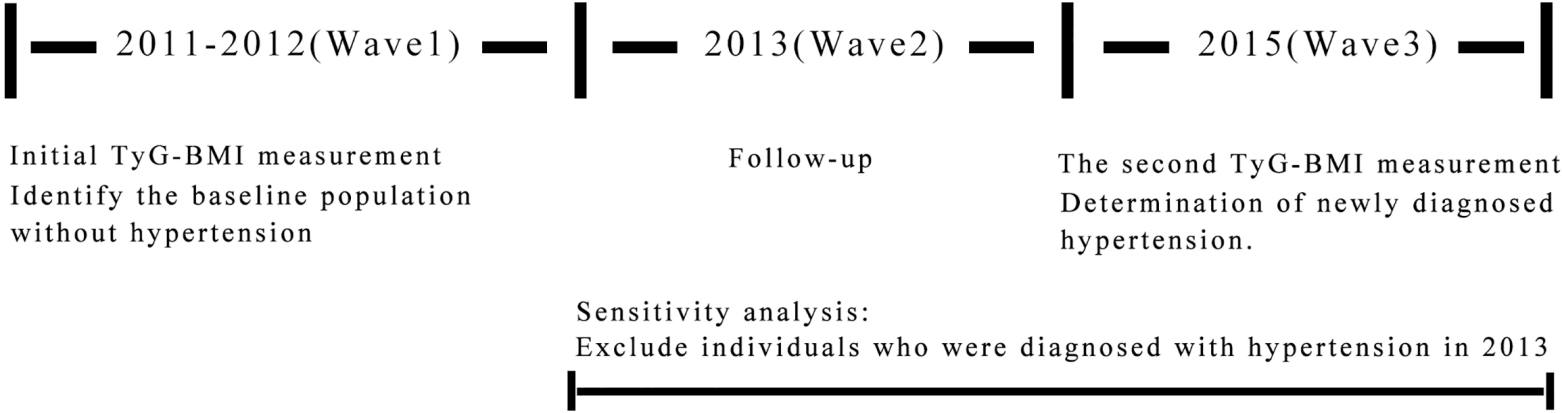
Timeline for inclusion and exclusion of study subjects.

**Fig 6:**
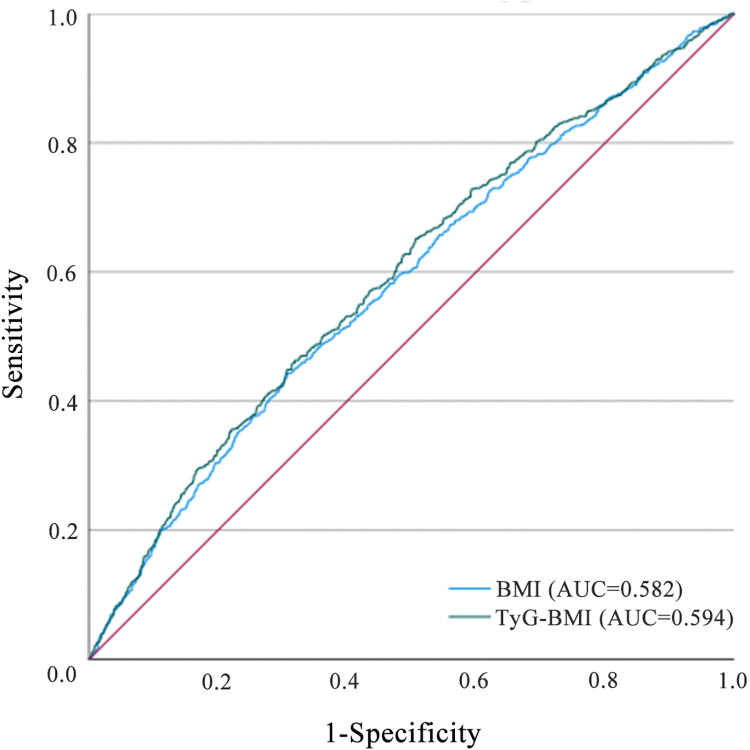
Comparison of predictive power among different indicators.

This study utilized CHARLS data from 2012 and 2015, employs the average TyG-BMI from two time points as the evaluation index and utilizes a cohort study design to investigate the association between this mid-term measure of TyG-BMI and the risk of hypertension. The results indicated that higher levels of average TyG-BMI (*Q*2-*Q*4) were associated with an increased risk of hypertension (*Q*2: *OR*=1.37, 95%*CI* = 1.04–1.80; *Q*3: *OR*=1.93, 95%*CI* = 1.46–2.56; *Q*4: *OR*=2.71, 95%*CI* = 2.01–3.66). Additionally, the results from the RCS model demonstrated a linear correlation between average TyG-BMI and the risk of hypertension, with a significant increase in hypertension risk observed when average TyG-BMI exceeded 613. Furthermore, subgroup analysis did not reveal any interactions between potential risk factors and the development of hypertension. Lastly, considering that TyG-BMI was jointly derived from TG, FPG, and BMI, this study, through WQS regression, identified BMI as the primary contributor to this association. Although the primary model adjusted for HDL-C and HbA1c to control for confounders, these factors may lie on the causal pathway between TyG-BMI and hypertension. Therefore, adjusting for such mediating variables might partially attenuate the true association. This attenuation is evident when comparing the effect sizes between the minimally adjusted (Model 1) and fully adjusted (Model 2) models. For example, the odds ratio for the highest quartile (*Q*4) of average TyG-BMI decreased from 2.93 (95% *CI*: 2.25–3.82) in Model 1 to 2.71 (95% *CI*: 2.01–3.66) in Model 2, following the inclusion of HDL-C and HbA1c. This consistent pattern of slight attenuation across quartiles (see S5 Table in S1 Appendix) indicates that a portion of the association between TyG-BMI and hypertension is likely mediated through pathways related to lipid and glucose metabolism. Furthermore, HDL-C and HbA1c reflect independent cardiovascular risk pathways that are not entirely captured by TyG-BMI. To balance these considerations, this study presents both fully adjusted and minimally adjusted models to ensure transparency Figs 5 and 6.

The results of this study indicate that average TyG-BMI is an independent risk factor for new onset hypertension. This finding is consistent with previous studies that have explored the association between TyG-BMI and hypertension risk based on measurements taken at a specific time point. A cross-sectional study conducted in Japan demonstrated that TyG-BMI was strongly correlated with hypertension risk; specifically, for every 10-unit increase in TyG-BMI, the risk of developing hypertension increased by 31% (*OR*=1.31, 95%*CI*: 1.25–1.37) [29]. Additionally, Lu Chen et al [30] investigated the relationship between TyG-BMI and hypertension in East Asian populations, revealing that TyG-BMI was independently associated with both prehypertension and hypertension. The findings from the 2017–2020 NHANES study indicate that for every 10-unit increase in TyG-BMI, the risk of hypertension increases by 4.3% (95% *CI*: 1.007–1.08). When TyG-BMI was categorized into quartiles, the association between TyG-BMI and the heightened risk of hypertension remained significant, demonstrating statistical significance across all models [31]. However, relying on a single measurement of TyG-BMI failed to account for its dynamic changes over time. Researchers have noted that blood parameters assessed at a single time point may be influenced by various factors, including diet and medications, leading to significant variability that may not accurately represent the subject’s long-term exposure [32]. This study found that individuals with high levels of average TyG-BMI had a 171% increased risk of developing hypertension compared to those with low levels (*OR*=2.71, 95%*CI* = 2.01–3.66). Therefore, it can be inferred from this study that prolonged exposure to elevated TyG-BMI poses a greater risk of hypertension than a single measurement. Furthermore, a sensitivity analysis that excluded hypertension cases from 2013 strengthened the causal inference by reducing reverse causality bias. Individuals diagnosed early may adopt dietary modifications, exercise interventions, or initiate antihypertensive or lipid-lowering treatments [33], all of which could independently reduce TyG-BMI. The persistence of the association, even after excluding these cases, indicates that cumulative TyG-BMI elevation is a risk factor for the incidence of hypertension, and the observed effect is not merely an artifact of post-diagnosis metabolic changes. This finding aligns with longitudinal evidence suggesting that insulin resistance predicts hypertension [34]; however, future research should directly track biomarker trajectories following diagnosis.

The mechanisms underlying long-term changes in TyG-BMI and their association with hypertension remain unclear, although insulin resistance may play a significant role. Insulin resistance is recognized as a risk factor for elevated blood pressure. When the body experiences prolonged insulin resistance, elevated insulin levels can enhance insulin-mediated glucose metabolism in neurons, stimulate central nervous pathways in the brainstem, and lead to increased sympathetic activity. This process results in heightened secretion of epinephrine and norepinephrine, which contribute to vascular smooth muscle thickening and lumen narrowing, ultimately resulting in increased blood pressure [35,36]. Additionally, some researchers suggest that insulin resistance may affect sodium and calcium ion pumps, leading to increased intracellular concentrations of sodium and calcium ions. This increase heightens the responsiveness of vascular smooth muscle to insulin-like growth factors, promotes further thickening of the vascular smooth muscle, stimulates sympathetic nerve activity, and enhances demethylation of adrenaline levels, thereby causing vasoconstriction and contributing to elevated blood pressure in patients [37].

WQS regression was employed to enhance the interpretability of TyG-BMI. The results indicated that in both 2012 and 2015, BMI was the primary contributor to the outcomes, suggesting that appropriate management of BMI ranges can mitigate the risk of hypertension. A study analyzing the NHANES non-diabetic and non-prediabetic population from 1999 to 2014 revealed that, compared to individuals with normal BMI, those classified as obese had a risk of insulin resistance that was 3.62 times higher, while the risk for those categorized as overweight was 3.19 times greater [38]. The duration of obesity is a significant risk factor for insulin resistance. Early insulin resistance may be countered by compensatory activation of pancreatic β-cell function; however, long-term persistent obesity can lead to pancreatic β-cell failure, potentially resulting in glucose metabolism disorders [39]. Research conducted by Baoyu Feng et al [40] demonstrated that as BMI increased, the risk of hypertension among overweight and obese individuals was 1.16 to 1.28 times greater than those of the normal weight group. Furthermore, the findings of a cross-sectional study involving 1.7 million participants indicated a positive linear relationship between BMI and both systolic and diastolic blood pressure. Specifically, for individuals with a BMI ranging from 18.5 to 30.0 kg/m², each 1 unit increase in BMI corresponded to an increase of 1.15 mmHg in systolic blood pressure and 0.75 mmHg in diastolic blood pressure [41]. The mechanisms through which obesity induces hypertension are complex. It is widely accepted in academic circles that this process involves the excessive activation of the sympathetic nervous system, stimulation of the renin-angiotensin-aldosterone system, alterations in adipose-derived cytokines, and insulin resistance, all of which contribute to the development of hypertension [42]. Although BMI emerged as the primary driver of the TyG-BMI index in our WQS analysis, this should not be interpreted as diminishing the value of this composite index. The TyG-BMI integrates complementary pathophysiological pathways of lipid metabolism (TG), glucose homeostasis (FPG), and obesity (BMI), thereby providing a more comprehensive measure of metabolic dysfunction. Moreover, previous studies have demonstrated that TyG-BMI outperforms BMI alone in predicting insulin resistance, metabolic syndrome, and cardiovascular events [13–16].

Notably, the data collection period for this study (2011−2015) predates the global COVID-19 pandemic (2020 onward). The exclusion of data from the pandemic era mitigates potential confounding effects arising from pandemic-related disruptions, such as lifestyle changes, limitations in healthcare access, and altered metabolic profiles due to lockdowns. This approach enhances the internal validity of our findings regarding the longitudinal association between average TyG-BMI and hypertension risk. However,This study has several limitations. (1) While numerous studies have demonstrated that TyG-BMI exhibits superior predictive performance compared to TyG in assessing insulin resistance, establishing a direct association between insulin resistance and hypertension risk necessitates comparison with a gold standard. However, CHARLS has not yet conducted a hyperinsulinemic euglycemic clamp trial, which precludes such comparisons in this study. (2) All participants in this study were of Chinese ethnicity, which may limit the generalizability of our findings to other ethnic populations. Significant variations in body composition, lipid metabolism, and genetic susceptibility to insulin resistance exist among individuals of different ethnicities and nationalities. Consequently, the risk threshold of cumulative mean TyG-BMI identified in our study may be most relevant to populations sharing similar genetic backgrounds, lifestyles, and environmental contexts as our study cohort. Therefore, it is recommended that future research validate these findings across diverse ethnicities, nationalities, and geographical settings to ascertain the universal applicability and optimal cutoff points of TyG-BMI in predicting hypertension. (3) Although we have accounted for potential confounding factors such as general information, behavioral habits, and medical history, it is possible that there remain unconsidered confounding variables that could influence the research outcomes. (4) Although the average TyG-BMI is widely adopted in cohort studies with limited time points, it remains an arithmetic mean rather than a time-weighted integral. (5) This study conducted only two measurements during the three-year follow-up period, which limited our ability to accurately describe the dynamic changes in individual TyG-BMI trajectories. This constitutes a significant limitation of the research.

## Conclusions

In summary, among middle-aged and elderly Chinese individuals, average TyG-BMI served as an independent risk factor for the development of new-onset hypertension, exhibiting a linear correlation between the two variables. Therefore, long-term monitoring of changes in TyG-BMI should have been a critical component of hypertension prevention strategies. Additionally, BMI emerged as the primary factor influencing the results, thereby elucidating the underlying mechanism associated with TyG-BMI.

## Supporting information

S1 Appendix Tables S1-S6 and figure S1-S2. (DOCX)

## Abbreviations

TyG: Triglyceride-glucose index
BMI: Body mass index
TyG-BMI: Triglyceride glucose-body mass index
CHARLS: China Health and Retirement Longitudinal Study
TG: Triglyceride
FPG: Fasting plasma glucose
TC: Total cholesterol
LDL-C: Low-density lipoprotein cholesterol
HDL-C: High-density lipoprotein cholesterol
HbA1c: Glycosylated hemoglobin
OR: Odds ratio
CI: Confidence interval
VIF: Variance inflation factor
BIC: Bayesian information criterion
AIC: Akaike information criterion
RCS: Restricted cubic spline
WQS: Weighted quantile sum
ROC: Receiver Operating Characteristic

## References

[1] Murray CJL, Aravkin AY, Zheng P, Abbafati C, Abbas KM, Abbasi-Kangevari M, et al. Global burden of 87 risk factors in 204 countries and territories, 1990–2019: a systematic analysis for the Global Burden of Disease Study 2019. The Lancet. 2020;396(10258):1223–49. doi: 10.1016/s0140-6736(20)30752-2 PMC7566194 33069327

[2] Zhou B, Carrillo-Larco RM, Danaei G, Riley LM, Paciorek CJ, Stevens GA, et al. Worldwide trends in hypertension prevalence and progress in treatment and control from 1990 to 2019: a pooled analysis of 1201 population-representative studies with 104 million participants. The Lancet. 2021;398(10304):957–80. doi: 10.1016/s0140-6736(21)01330-1 PMC8446938 34450083

[3] Mills KT, Bundy JD, Kelly TN, Reed JE, Kearney PM, Reynolds K, et al. Global disparities of hypertension prevalence and control: a systematic analysis of population-based studies from 90 countries. Circulation. 2016;134(6):441–50. doi: 10.1161/CIRCULATIONAHA.115.018912 27502908 PMC4979614

[4] Zhang M, Shi Y, Zhou B, Huang Z, Zhao Z, Li C, et al. Prevalence, awareness, treatment, and control of hypertension in China, 2004-18: findings from six rounds of a national survey. BMJ. 2023;380:e071952. doi: 10.1136/bmj-2022-071952 36631148 PMC10498511

[5] Yuan L, Chen M. Insulin resistance, hyperinsulinaemia and hypertension. Chinese Journal of Hypertension. 2020;28(07):616–20. doi: 10.16439/j.cnki.1673-7245.2020.07.004

[6] Chinese Diabetes Society Insulin Resistance Study Group. Expert guidance on insulin resistance assessment methods and applications. Chinese J Diabetes Mellitus. 2018;10(6):377–85. doi: 10.3760/cma.j.issn.1674-5809.2018.06.001

[7] Huang R, Wang Z, Chen J, Bao X, Xu N, Guo S, et al. Prognostic value of triglyceride glucose (TyG) index in patients with acute decompensated heart failure. Cardiovasc Diabetol. 2022;21(1). doi: 10.1186/s12933-022-01507-7 PMC9158138 35641978

[8] Zhou Z, Liu Q, Zheng M, Zuo Z, Zhang G, Shi R, et al. Comparative study on the predictive value of TG/HDL-C, TyG and TyG-BMI indices for 5-year mortality in critically ill patients with chronic heart failure: a retrospective study. Cardiovasc Diabetol. 2024;23(1). doi: 10.1186/s12933-024-02308-w PMC11191322 38902757

[9] Toro-Huamanchumo CJ, Urrunaga-Pastor D, Guarnizo-Poma M, Lazaro-Alcantara H, Paico-Palacios S, Pantoja-Torres B, et al. Triglycerides and glucose index as an insulin resistance marker in a sample of healthy adults. Diabetes & Metabolic Syndrome: Clinical Research & Reviews. 2019;13(1):272–7. doi: 10.1016/j.dsx.2018.09.010 30641711

[10] Su Q, Zang L. Insulin resistance: history, mechanisms and management. Chinese Journal of Diabetes Mellitus. 2023;15(1):6–13. doi: 10.3760/cma.j.cn115791-20220905-00447

[11] Bala C, Gheorghe-Fronea O, Pop D, Pop C, Caloian B, Comsa H, et al. The association between six surrogate insulin resistance indexes and hypertension: a population-based study. Metab Syndr Relat Disord. 2019;17(6):328–33. doi: 10.1089/met.2018.0122 31034338

[12] Ramírez-Vélez R, Pérez-Sousa MÁ, González-Ruíz K, Cano-Gutierrez CA, Schmidt-RioValle J, Correa-Rodríguez M, et al. Obesity- and lipid-related parameters in the identification of older adults with a high risk of prediabetes according to the american diabetes association: an analysis of the 2015 health, well-being, and aging study. Nutrients. 2019;11(11):2654. doi: 10.3390/nu11112654 31689977 PMC6893527

[13] Lim J, Kim J, Koo SH, Kwon GC. Comparison of triglyceride glucose index, and related parameters to predict insulin resistance in Korean adults: an analysis of the 2007-2010 Korean National health and nutrition examination survey. PLoS One. 2019;14(3):e0212963. doi: 10.1371/journal.pone.0212963 30845237 PMC6405083

[14] Raimi TH, Dele-Ojo BF, Dada SA, Fadare JO, Ajayi DD, Ajayi EA, et al. Triglyceride-glucose index and related parameters predicted metabolic syndrome in Nigerians. Metab Syndr Relat Disord. 2021;19(2):76–82. doi: 10.1089/met.2020.0092 33170086 PMC7929914

[15] Dang K, Wang X, Hu J, Zhang Y, Cheng L, Qi X, et al. The association between triglyceride-glucose index and its combination with obesity indicators and cardiovascular disease: NHANES 2003-2018. Cardiovasc Diabetol. 2024;23(1):8. doi: 10.1186/s12933-023-02115-9 38184598 PMC10771672

[16] Dou J, Guo C, Wang Y, Peng Z, Wu R, Li Q, et al. Association between triglyceride glucose-body mass and one-year all-cause mortality of patients with heart failure: a retrospective study utilizing the MIMIC-IV database. Cardiovasc Diabetol. 2023;22(1):309. doi: 10.1186/s12933-023-02047-4 37940979 PMC10634170

[17] Miao H, Zhou Z, Yang S, Zhang Y. The association of triglyceride-glucose index and related parameters with hypertension and cardiovascular risk: a cross-sectional study. Hypertens Res. 2023;47(4):877–86. doi: 10.1038/s41440-023-01502-9 38012411

[18] Chen J, Chen J, Hu W, Zhang Q, Lu S. Correlation analysis of TyG and TyG-BMI with hypertension. Modern Medical Journal. 2024;52(03):379–84. doi: 10.3969/j.issn.1671-7562.2024.03.008

[19] Deng D, Chen C, Wang J, Luo S, Feng Y. Association between triglyceride glucose-body mass index and hypertension in Chinese adults: a cross-sectional study. J Clin Hypertens (Greenwich). 2023;25(4):370–9. doi: 10.1111/jch.14652 36929716 PMC10085812

[20] Zhao Y, Strauss J, Yang G, Giles J, Hu P, Hu Y. China health and retirement longitudinal study: 2011-2012 national baseline user’s guide. National School of Development: Peking University; 2013.

[21] Zhao Y, Hu Y, Smith JP, Strauss J, Yang G. Cohort profile: the China Health and Retirement Longitudinal Study (CHARLS). Int J Epidemiol. 2014;43(1):61–8. doi: 10.1093/ije/dys203 23243115 PMC3937970

[22] Chen X, Crimmins E, Hu PP, Kim JK, Meng Q, Strauss J, et al. Venous blood-based biomarkers in the China health and retirement longitudinal study: rationale, design, and results from the 2015 wave. Am J Epidemiol. 2019;188(11):1871–7. doi: 10.1093/aje/kwz170 31364691 PMC6825825

[23] Huo R-R, Zhai L, Liao Q, You X-M. Changes in the triglyceride glucose-body mass index estimate the risk of stroke in middle-aged and older Chinese adults: a nationwide prospective cohort study. Cardiovasc Diabetol. 2023;22(1):254. doi: 10.1186/s12933-023-01983-5 37716947 PMC10505325

[24] Cui H, Liu Q, Wu Y, Cao L. Cumulative triglyceride-glucose index is a risk for CVD: a prospective cohort study. Cardiovasc Diabetol. 2022;21(1):22. doi: 10.1186/s12933-022-01456-1 35144621 PMC8830002

[25] Joint Committee for Guideline Revision. 2018 Chinese guidelines for prevention and treatment of hypertension-A report of the revision committee of Chinese guidelines for prevention and treatment of hypertension. J Geriatr Cardiol. 2019;16(3):182–241. doi: 10.11909/j.issn.1671-5411.2019.03.014 31080465 PMC6500570

[26] Li J-J, Zhao S-P, Zhao D, Lu G-P, Peng D-Q, Liu J, et al. 2023 China guidelines for lipid management. J Geriatr Cardiol. 2023;20(9):621–63. doi: 10.26599/1671-5411.2023.09.008 37840633 PMC10568545

[27] Jia W, Weng J, Zhu D, Ji L, Lu J, Zhou Z, et al. Standards of medical care for type 2 diabetes in China 2019. Diabetes Metab Res Rev. 2019;35(6):e3158. doi: 10.1002/dmrr.3158 30908791

[28] Tanner EM, Bornehag C-G, Gennings C. Repeated holdout validation for weighted quantile sum regression. MethodsX. 2019;6:2855–60. doi: 10.1016/j.mex.2019.11.008 31871919 PMC6911906

[29] Huang X, He J, Wu G, Peng Z, Yang B, Ye L. TyG-BMI and hypertension in Normoglycemia subjects in Japan: a cross-sectional study. Diabetes and Vascular Disease Research. 2023;20(3). doi: 10.1177/14791641231173617 PMC10201169 37209031

[30] Chen L, He L, Zheng W, Liu Q, Ren Y, Kong W, et al. High triglyceride glucose-body mass index correlates with prehypertension and hypertension in east Asian populations: a population-based retrospective study. Front Cardiovasc Med. 2023;10. doi: 10.3389/fcvm.2023.1139842 PMC10166815 37180805

[31] Zhao L, Zheng L, Wang R, Gong X, Wu Y, Han S, et al. Association between triglyceride glucose combined with body mass index and hypertension in the NHANES 2017 to 2020. Sci Rep. 2025;15(1):9092. doi: 10.1038/s41598-025-93723-w 40097561 PMC11914623

[32] Liu Z, Zhang L, Wang L, Li K, Fan F, Jia J, et al. The predictive value of cumulative atherogenic index of plasma (AIP) for cardiovascular outcomes: a prospective community-based cohort study. Cardiovasc Diabetol. 2024;23(1):264. doi: 10.1186/s12933-024-02350-8 39026310 PMC11264486

[33] Altmaier E, Fobo G, Heier M, Thorand B, Meisinger C, Römisch-Margl W, et al. Metabolomics approach reveals effects of antihypertensives and lipid-lowering drugs on the human metabolism. Eur J Epidemiol. 2014;29(5):325–36. doi: 10.1007/s10654-014-9910-7 24816436 PMC4050296

[34] Hou X-Z, Lv Y-F, Li Y-S, Wu Q, Lv Q-Y, Yang Y-T, et al. Association between different insulin resistance surrogates and all-cause mortality in patients with coronary heart disease and hypertension: NHANES longitudinal cohort study. Cardiovasc Diabetol. 2024;23(1):86. doi: 10.1186/s12933-024-02173-7 38419039 PMC10903030

[35] da Silva AA, do Carmo JM, Li X, Wang Z, Mouton AJ, Hall JE. Role of hyperinsulinemia and insulin resistance in hypertension: metabolic syndrome revisited. Can J Cardiol. 2020;36(5):671–82. doi: 10.1016/j.cjca.2020.02.066 32389340 PMC7219403

[36] Ran R, Tan W. Research progress on triglyceride glucose index in hypertension. Chinese J Difficult Complicated Cases. 2024;23(05):617-620 630. doi: 10.3969/j.issn.1671-6450.2024.05.022

[37] Luo J. Research progress on the relationship between insulin resistance and cardiovascular disease in type 2 diabetes mellitus. Journal of Public Health and Preventive Medicine. 2023;34(05):125–8. doi: 10.3969/j.issn.1006-2483.2023.05.028

[38] Caporaso NE, Jones RR, Stolzenberg-Solomon RZ, Medgyesi DN, Kahle LL, Graubard BI. Insulin resistance in healthy u.s. adults: findings from the national health and nutrition examination survey (NHANES). Cancer Epidemiol Biomarkers Prev. 2020;29(1):157–68. doi: 10.1158/1055-9965.EPI-19-0206 31641012

[39] Kim K-S, Lee Y-M, Lee I-K, Kim D-J, Jacobs DR Jr, Lee D-H. Paradoxical associations of insulin resistance with total and cardiovascular mortality in humans. J Gerontol A Biol Sci Med Sci. 2015;70(7):847–53. doi: 10.1093/gerona/glu194 25326285

[40] Feng BY, Chen JC, Li Y, Huang JF, Li JX, Zhao LC, et al. Relationship between overweight/obesity and hypertension among adults in China: a prospective study. Zhonghua Liu Xing Bing Xue Za Zhi. 2016;37(5):606–11. doi: 10.3760/cma.j.issn.0254-6450.2016.05.004 27188347

[41] Linderman GC, Lu J, Lu Y, Sun X, Xu W, Nasir K, et al. Association of body mass index with blood pressure among 1.7 million chinese adults. JAMA Netw Open. 2018;1(4):e181271. doi: 10.1001/jamanetworkopen.2018.1271 PMC6324286 30646115

[42] Shariq OA, McKenzie TJ. Obesity-related hypertension: a review of pathophysiology, management, and the role of metabolic surgery. Gland Surg. 2020;9(1):80–93. doi: 10.21037/gs.2019.12.03 32206601 PMC7082272

