# Supplementary material for: Association between average triglyceride glucose-body mass index and risk of hypertension in middle-aged and elderly Chinese: A study based on Chinese CHARLS cohort data
Source: PLoS One. 2025 Dec 4;20(12):e0337710. doi: 10.1371/journal.pone.0337710 (PMC12677574; doi:10.1371/journal.pone.0337710)
Supplement: S1 Appendix — (DOCX) [file pone.0337710.s001.docx]

Table S1 Collinearity diagnosis

| Model | Unstandardized coefficients | | Standardized coefficients | *t* | *P* | Collinearity Statistics | |
| --- | --- | --- | --- | --- | --- | --- | --- |
|  | *B* | standard error | Beta |  |  | Tolerances | VIF |
| Constant | -0.312 | 0.120 |  | -2.602 | 0.009^**^ |  |  |
| Sex | -0.059 | 0.025 | -0.068 | -2.333 | 0.020^*^ | 0.394 | 2.540 |
| Age | 0.005 | 0.001 | 0.094 | 4.491 | <0.001^***^ | 0.768 | 1.302 |
| Residence | -0.031 | 0.024 | -0.025 | -1.300 | 0.194 | 0.900 | 1.112 |
| Education | -0.015 | 0.012 | -0.027 | -1.251 | 0.211 | .723 | 1.383 |
| Marital status | 0.028 | 0.030 | 0.018 | 0.960 | 0.337 | 0.943 | 1.060 |
| Health status | 0.026 | 0.012 | 0.041 | 2.171 | 0.030^*^ | 0.959 | 1.043 |
| Smoking status | 0.015 | 0.024 | 0.018 | 0.650 | 0.516 | 0.464 | 2.156 |
| Drinking status | -0.033 | 0.020 | -0.034 | -1.611 | 0.107 | 0.781 | 1.280 |
| HDL-C | 0.004 | 0.001 | 0.123 | 4.822 | <0.001^***^ | 0.524 | 1.909 |
| LDL-C | 0.001 | 0.001 | 0.065 | 1.562 | 0.118 | 0.195 | 5.126 |
| TC | -0.001 | 0.001 | -0.066 | -1.394 | 0.164 | 0.153 | 6.548 |
| History of dyslipidemia | 0.107 | 0.020 | 0.122 | 5.401 | <0.001^***^ | 0.659 | 1.518 |
| History of diabetes | 0.086 | 0.032 | 0.060 | 2.671 | 0.008^**^ | 0.678 | 1.475 |
| HbAlc | 0.000 | 0.011 | -0.001 | -0.028 | 0.978 | 0.667 | 1.499 |
| Average TyG-BMI quartiles | 0.060 | 0.009 | 0.156 | 6.898 | <0.001^***^ | 0.666 | 1.501 |

Table S2 Restricted Cubic Spline Node Selection

| Node | AIC | BIC |
| --- | --- | --- |
| 3 | 3003.040 | 3103.969 |
| 4 | 3004.227 | 3111.093 |
| 5 | 3006.063 | 3118.867 |
| 6 | 3007.680 | 3126.421 |
| 7 | 3007.245 | 3131.922 |

TableS3 Association of average TyG-BMI with hypertension incidence when treating

average TyG-BMI as a continuous variable

| Average TyG-BMI | Unadjusted | *P* | Model 1^a^ | *P* | Model 2^b^ | *P* |
| --- | --- | --- | --- | --- | --- | --- |
|  | *OR*（95%*CI*） |  | *OR*（95%*CI*） |  | *OR*（95%*CI*） |  |
| Per 1 *SD* | 1.35 (1.24,1.47) | <0.001^***^ | 1.48(1.36,1.62) | <0.001^***^ | 1.43 (1.29,1.58) | <0.001^***^ |

a: Adjust sex, age, residence, education, marital status, health status, smoking status, and drinking status.

b: Adjust the variables in Model 1: history of dyslipidemia, history of diabetes, HDL-C, and HbA1c.

TableS4 Association between average TyG-BMI and the incidence of hypertension after

excluding patients in 2013

|  | Unadjusted | *P* | Model 1^a^ | *P* | Model 2^b^ | *P* |
| --- | --- | --- | --- | --- | --- | --- |
|  | *OR*（95%*CI*） |  | *OR*（95%*CI*） |  | *OR*（95%*CI*） |  |
| Average TyG-BMI |  |  |  |  |  |  |
| *Q*1 | Reference |  | Reference |  | Reference |  |
| *Q*2 | 0.95 (0.65,1.38) | 0.785 | 1.02 (0.70,1.49) | 0.934 | 1.07 (0.72,1.58) | 0.743 |
| *Q*3 | 1.54 (1.01,2.18) | 0.014^*^ | 1.80 (1.25,2.58) | 0.002^**^ | 1.79 (1.22,2.63) | 0.003^**^ |
| *Q*4 | 2.09 (1.50,2.92) | <0.001^***^ | 2.56 (1.80,3.65) | <0.001^***^ | 2.41 (1.62,3.61) | <0.001^***^ |

a: Adjust sex, age, residence, education, marital status, health status, smoking status, and drinking status.

b: Adjust the variables in Model 1: history of dyslipidemia, history of diabetes, HDL-C, and HbA1c.

Table S5 Exploring the relationship between average TyG-BMI and the risk of hypertension incidence

in the minimally adjusted model

|  | Unadjusted | *P* | Model 1^a^ | *P* | Model 2^b^ | *P* |
| --- | --- | --- | --- | --- | --- | --- |
|  | *OR*（95%*CI*） |  | *OR*（95%*CI*） |  | *OR*（95%*CI*） |  |
| Average TyG-BMI |  |  |  |  |  |  |
| *Q*1 | Reference |  | Reference |  | Reference |  |
| *Q*2 | 1.19 (0.91-1.54) | 0.201 | 1.32 (1.01-1.72) | 0.044 | 1.37 (1.04-1.80) | 0.026^*^ |
| *Q*3 | 1.56 (1.21-2.01) | <0.001^***^ | 1.93 (1.48-2.51) | <0.001^***^ | 1.93 (1.46-2.56) | <0.001^***^ |
| *Q*4 | 2.23 (1.75-2.85) | <0.001^***^ | 2.93 (2.25-3.82) | <0.001^***^ | 2.71 (2.01-3.66) | <0.001^***^ |

a: Adjust sex, age, residence, education, marital status, health status, smoking status, and drinking status.

b: Adjust the variables in Model 1: history of dyslipidemia, history of diabetes.

TyG-BMI, Triglyceride glucose-body mass index; OR, Odds ratio; CI, Confidence interval; Q1, Quartile 1; Q2, Quartile 2; Q3, Quartile 3; Q4, Quartile 4.

**P* < 0.05, ***P* < 0.01, ****P* < 0.001.

Table S6 Exploring the relationship between mean TyG-BMI and hypertension risk in the fully adjusted model.

|  | Unadjusted | *P* | Model 1^a^ | *P* | Model 2^b^ | *P* |
| --- | --- | --- | --- | --- | --- | --- |
|  | *OR*（95%*CI*） |  | *OR*（95%*CI*） |  | *OR*（95%*CI*） |  |
| Average TyG-BMI |  |  |  |  |  |  |
| *Q*1 | Reference |  | Reference |  | Reference |  |
| *Q*2 | 1.19 (0.91-1.54) | 0.201 | 1.32 (1.01-1.72) | 0.044^*^ | 1.36 (1.03-1.79) | 0.031^*^ |
| *Q*3 | 1.56 (1.21-2.01) | <0.001^***^ | 1.93 (1.48-2.51) | <0.001^***^ | 1.93 (1.45-2.56) | <0.001^***^ |
| *Q*4 | 2.23 (1.75-2.85) | <0.001^***^ | 2.93 (2.25-3.82) | <0.001^***^ | 2.79 (2.05-3.80) | <0.001^***^ |

a: Adjust sex, age, residence, education, marital status, health status, smoking status, and drinking status.

b: Adjust the variables in Model 1: history of dyslipidemia, history of diabetes, HDL-C, LDL-C, TC and HbA1c.

TyG-BMI, Triglyceride glucose-body mass index; OR, Odds ratio; CI, Confidence interval; Q1, Quartile 1; Q2, Quartile 2; Q3, Quartile 3; Q4, Quartile 4; HbA1c, Glycosylated hemoglobin; HDL-C, High-density lipoprotein cholesterol; LDL-C, Low-density lipoprotein cholesterol; TC, Total cholesterol.

**P* < 0.05, ***P* < 0.01, ****P* < 0.001.

**Figure S1 Timeline for Inclusion and Exclusion of Study Subjects.** TyG-BMI, Triglyceride glucose-body mass index


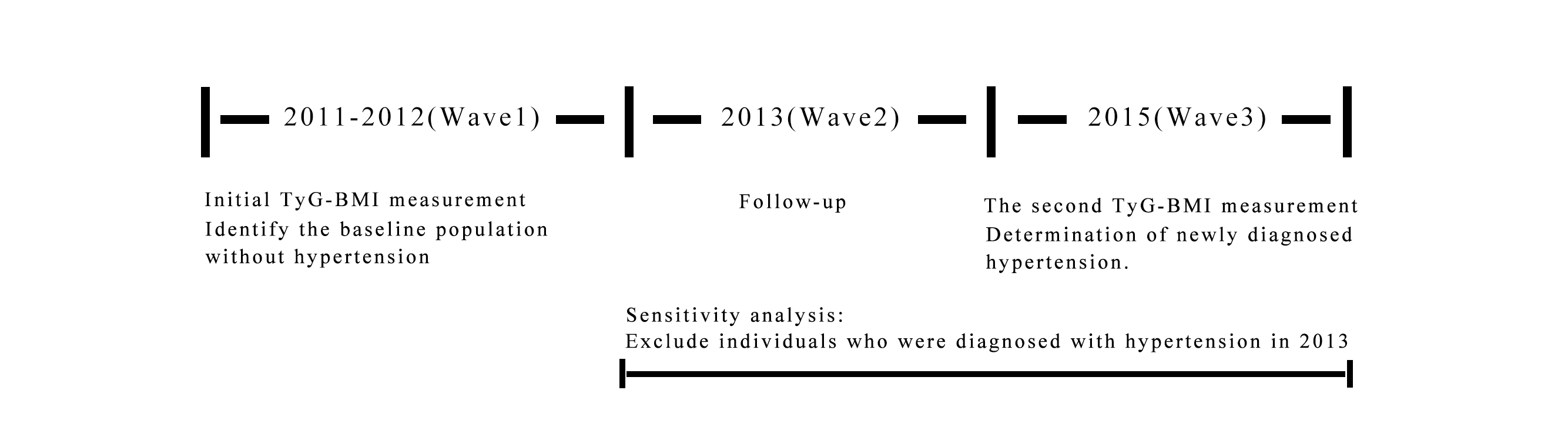

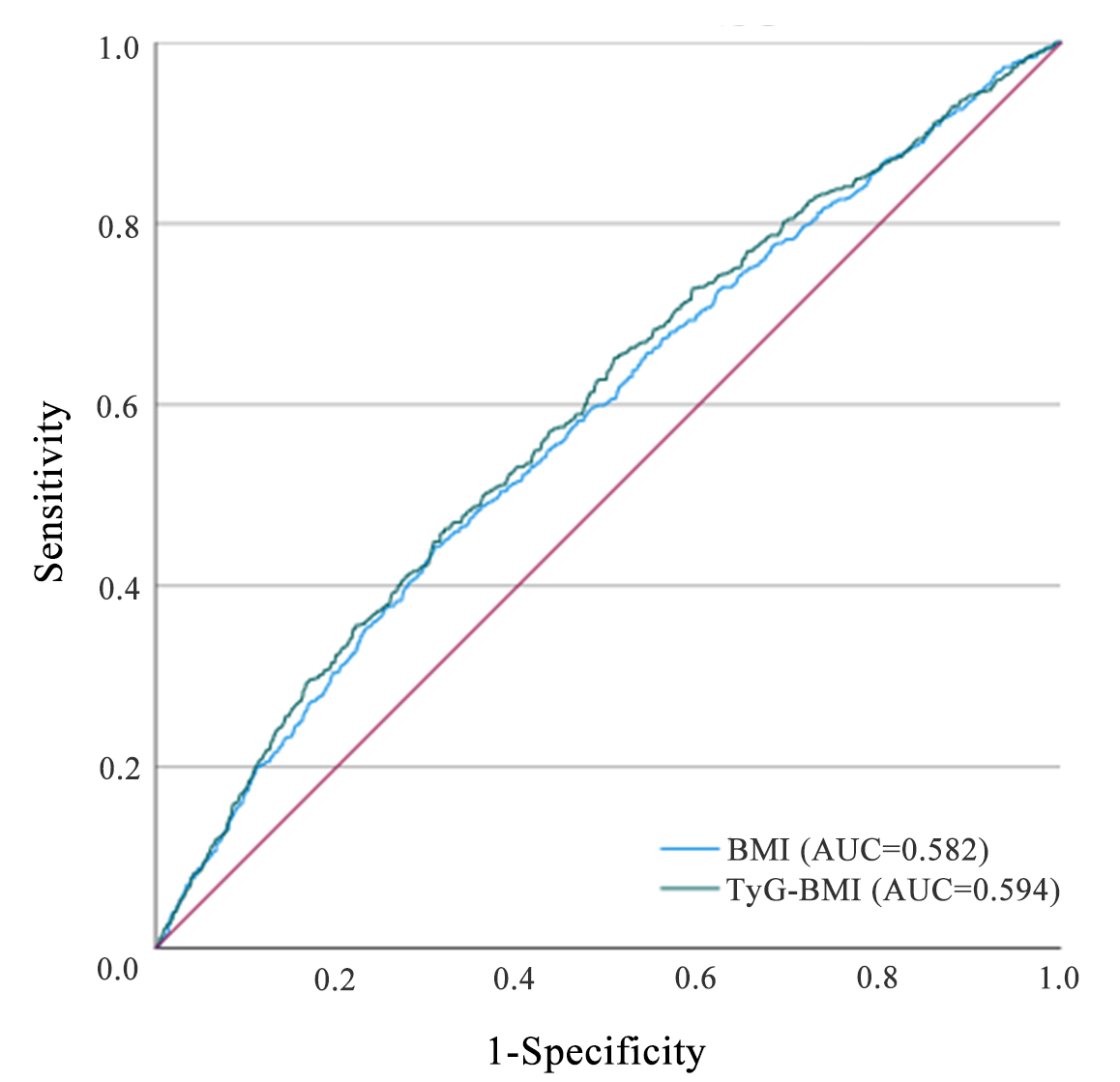


**Figure S2 Comparison of Predictive Power Among Different Indicators.** TyG-BMI, Triglyceride glucose-body mass index; BMI, body mass index; AUC, Area Under Curve.
